# Effect of High-Fat Diet on Cardiac Metabolites and Implications for Vulnerability to Ischemia and Reperfusion Injury

**DOI:** 10.3390/cells14171329

**Published:** 2025-08-28

**Authors:** Jihad S. Hawi, Katie L. Skeffington, Megan Young, Massimo Caputo, Raimondo Ascione, M-Saadeh Suleiman

**Affiliations:** 1 Department of Anatomy, Cell Biology, and Physiological Sciences, Faculty of Medicine, American University of Beirut, Beirut 11-0236, Lebanon; 2 Bristol Medical School, University of Bristol, Bristol BS2 8HW, UK; m.e.young@qmul.ac.uk (M.Y.); m.caputo@bristol.ac.uk (M.C.); r.ascione@bristol.ac.uk (R.A.); m.s.suleiman@bristol.ac.uk (M.-S.S.)

**Keywords:** ischemic reperfusion injury, cardiac metabolites, taurine, amino acids, high-fat diet, adenine nucleotide

## Abstract

Previous work has shown that mouse models fed a non-obesogenic high-fat diet have preserved cardiac function and no obesity-associated comorbidities such as diabetes. However, they do suffer increased cardiac vulnerability to ischemic reperfusion (I/R) injury, which has been attributed to changes in Ca^2+^ handling, oxidative stress, and mitochondrial transition pore activity. However, there have been no studies investigating the involvement of metabolites. Wild-type mice were fed either a control or a non-obesogenic high-fat diet for ~26 weeks. Key cardiac metabolites were extracted from freshly excised hearts and from hearts exposed to 30 min global ischemia followed by 45 min reperfusion. The extracted metabolites were measured using commercially available kits and HPLC. Hemodynamic cardiac function was monitored in Langendorff perfused hearts. Levels of energy-rich phosphates and related metabolites were similar for both hearts fed a control or a high-fat diet. However, the high-fat diet decreased cardiac glycogen and increased cardiac lactate, hypoxanthine, alanine, and taurine levels. Langendorff perfused hearts from the high-fat diet group suffered more ischemic stress during ischemia, as shown by the significantly shorter time needed for onset and for reaching maximal ischemic (rigor) contracture. Following I/R, there was a significant decrease in myocardial adenine nucleotides and a significant increase in the levels of alanine and purines for both groups. Most of the principal amino acids tended to fall during I/R. Hearts from mice fed a high-fat diet showed more changes during I/R in markers of energetics (phosphorylation potential and energy charge), metabolic stress (lactate), and osmotic stress (taurine). This study suggests that cardiac metabolic changes due to high-fat diet feeding, independent of obesity-related comorbidities, are responsible for the marked metabolic changes and the increased vulnerability to I/R.

## 1. Introduction

Experimental models of obesity in mice are expected to show a significant increase in body weight of approximately 30% or more [1,2]. This is commonly achieved by the use of a high-fat diet with high sucrose and low starch content [3,4,5], and is associated with the development of diabetes and cardiac dysfunction [3,6,7], with little change in blood pressure [8,9].

In contrast, wild-type mice fed a non-obesogenic high-fat diet (essentially containing fat lard, starch, and cholesterol) are not only resistant to coronary disease, but also do not develop diabetes, hypertension, atherosclerosis, cardiac hypertrophy, or functional impairment. However, atherosclerosis is found to develop in transgenic (e.g., apolipoprotein E knockout) mice fed this diet, as well as in mice undergoing further interventions, such as extended feeding [4,10].

It is widely accepted that obese rodent models demonstrate increased vulnerability of the myocardium to ischemic reperfusion (I/R) injury, with obesity and associated comorbidities implicated as responsible for the change in vulnerability [11,12,13,14,15]. Such vulnerability to I/R injury is independent of whether the solutions perfusing the heart contain lipids or not [16]. However, it has also been demonstrated that, despite lacking obesity-associated comorbidities (diabetes, cardiac hypertrophy, cardiac dysfunction, or atherosclerosis), wild-type mice fed a non-obesogenic high-fat diet still exhibit increased vulnerability to I/R, indicating that hyperlipidaemia alone is also responsible for augmenting I/R vulnerability [4,10]. Alterations in oxidative state, mitochondria, and Ca^2+^ handling were detected in these hearts; these factors likely contribute to the high-fat diet-induced vulnerability to I/R. Although these factors mediate I/R injury, the extent of their changes and/or contribution to myocardial damage will depend on the initial severity of metabolic ischemic alterations (e.g., ATP, lactate, and osmolytes) that occur prior to the involvement of triggers of death of cardiomyocytes (e.g., Ca^2+^ overload and oxidative stress). To date, no work has been performed investigating the effect of a non-obesogenic high-fat diet on key cardiac metabolites, including adenine nucleotides, purines (markers of cardiac energetics), taurine, protein amino acids (which have important roles as osmolytes and in metabolism), lactate, and glycogen. The goal of this study was therefore to ascertain the effect of a non-obesogenic high-fat diet on the levels of these key metabolites.

## 2. Materials and Methods

### 2.1. Animals

Animal procedures were as described in our previous publication [10]. Briefly, the animals (C57BL/6.129 male mice) were bred within the Animal Unit of the University of Bristol. All animal procedures were performed in accordance with the guidelines and regulations set at the time by the University of Bristol and the Home Office. At approximately 8 weeks of age, animals were either fed a Western-type high-fat diet (Special Diets Services, UK: 821424) or maintained on a normal rodent diet (Special Diets Services, UK, code: 801900) for 26 weeks with free access to water. This duration was chosen to correspond with previous work involving transgenic mice [10].

The content of the high-fat diet has been previously reported [4]. Briefly, it consisted of 45% calories from fat, 18% calories from protein, and 37% calories from carbohydrate, which was mostly starch. The dietary fat was primarily from lard (21%) and consisted of a mixture of saturated (44%) and mono- (43%) and poly-unsaturated (13%) fatty acids. The high-fat diet also contained 0.17% calories from cholesterol and low sucrose content. This diet is known to promote atherosclerosis in transgenic mice models without inducing significant body weight gain [10]. In contrast, and when fed to wild-type mice, this diet is not associated with cardiovascular disease or obesity, despite being hypercholesterolemic and hyperlipidaemic [4,10]. The standard chow diet fed to the control group consisted of 13% calories from fat, 22% calories from protein, and 65% calories from carbohydrate.

### 2.2. Functional Measurements of Isolated Hearts

**Figure 1:**
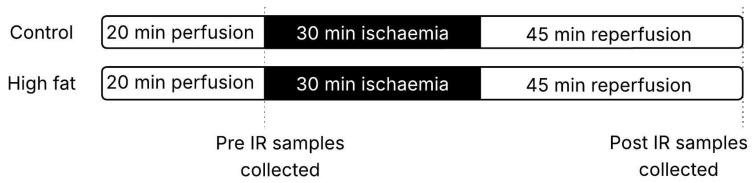
Perfusion protocol, showing times of collection of pre-IR and post-IR samples.

Animals were killed by cervical dislocation, and the hearts were excised and rinsed in ice-cold Krebs’ solution (in mmol/L): 120 NaCl, 25 NaHCO_3_, 11 glucose, 1.2 KH_2_PO_4_, 1.2 MgSO_4_, 4.8 KCl, 1.2 CaCl_2_, 2 Na-pyruvate. The hearts were immediately cannulated via the aorta and perfused retrogradely with Krebs’ solution on a Langendorff set-up (ADInstruments, Oxford, UK). The perfusion pressure was kept constant via a MacLab pump controller at 65 mmHg, and the heart and solutions were kept at 37 °C via immersion in perfusion solution and water jackets, respectively. Air with 95% oxygen was used to bubble the solution reservoirs. A cling film balloon filled with water was inserted into the left ventricle, connected via a pressure transducer to a MacLab apparatus and used to measure left ventricular developed pressure (LVDP) and computed heart rate. Haemodynamic recordings were made using PowerLab Chart 5 software (ADInstruments). Hearts were allowed to stabilize for approximately 20 min before either being collected (see below and Figure 1) or exposed to global, normothermic ischemia for 30 min (by turning off the perfusate), followed by 45 min reperfusion. This ischemic duration was chosen as 35 min ischemia was associated with very poor recovery during reperfusion.

### 2.3. Collection of Myocardial Tissue

Ventricular tissue was collected from hearts following 20 min perfusion and stabilization (pre-IR samples) and at the end of I/R (30 min ischemia and 45 min reperfusion) protocol (post-IR samples), both from control and high-fat diet mice (Figure 1). Tissue was snap-frozen in liquid nitrogen and stored at −80 °C. Subsequently, ventricular tissue was used to extract and measure protein and metabolites.

### 2.4. Extraction and Measurement of Cardiac Metabolites

Whole ventricular myocardial tissue samples were crushed in liquid nitrogen and added to an Eppendorff tube containing 500 µL of 4.8% PCA. Tubes were vortexed, and then spun at 2880× *g* for 10 min at 4 °C. The supernatant (300 µL) and K_2_CO_3_ (300 µL) were pipetted into a further set of labeled tubes, vortexed, and spun at 2880× *g* for 10 min at 4 °C. A 300 µL aliquot of supernatant was removed and stored at minus 20 °C for adenine and purine nucleotides and lactate measurement. A further 100 µL of supernatant was removed, added to 100 µL HCl, vortexed, and then dried in a savant to a pressure of less than 400 U and stored at minus 20 °C for amino acid measurement. The remaining pellet after the supernatant had been removed was dried using blotting paper, 500 µL double distilled water was added, and it was then stored at minus 20 °C for protein determination. Myocardial protein and lactate concentrations were then measured with the use of commercially available kits (Sigma, Gillingham, UK); amino acid and nucleotide contents of myocardial tissue were analyzed using high-performance liquid chromatography (HPLC). The two HPLC columns used were designed and calibrated for the measurement of amino acids and ATP-related metabolites only. Lactate, amino acids, and nucleotides were expressed in nmol/mg protein as described previously [17]. It is important to note, when comparing with other studies, that HPLC measures total ADP (bound and free), while other methods may only measure free ADP. Energy charge and phosphorylation potential (ATP/AMP) were calculated as below:

(1) $$Energy\;charge=\;\frac{ATP+0.5ADP}{ATP+ADP+AMP}$$

(2) $$Phosphorylation\;potential=\frac{ATP}{AMP}$$

Both freshly excised hearts and hearts exposed to 30 min of ischemia followed by 45 min of reperfusion, taken from animals on a control or high-fat diet, were used.

### 2.5. Extraction and Measurement of Cardiac Glycogen

An additional group of animals was used to extract and measure glycogen content in hearts from mice fed a normal or high-fat diet. Frozen ventricular tissue (50 mg) was crushed in liquid nitrogen and 0.2 mL of 30% KOH was added and mixed until dissolved. Samples were then left on a heating block at 105 °C for 1 h. After this period, tubes were cooled to 0 °C on ice. Then, 0.1 mL of 2% Na_2_SO_4_ was added and mixed until dissolved; 100% ethanol was then added to give a final concentration of 75% *v*/*v* (assuming 1 g tissue = 1 mL). Samples were then left in the fridge at 4 °C overnight. Samples were spun at 13,000 rpm for 2 min at room temperature and the supernatant removed. They were then re-suspended in 80% ethanol, spun again, and the supernatant once again removed. Samples were left on a heating block at 40 °C overnight. The following solutions were then added and mixed until dissolved: 0.25 mL of 1 mol/L sodium acetate/acetic acid; 0.1 mL 500 μL/mL amyloglucosidase (500 μL amyloglucosidase in 1 mL sodium acetate/acetic acid); and 0.75 mL water. Samples were incubated in a water bath at 37 °C for 1 h. A further 0.2 mL water was added, the samples were spun at 13,000 rpm for 2 min, and the supernatant was removed and assayed for total glucose content using a commercially available kit (Sigma, UK).

### 2.6. Statistical Analysis

All data are expressed as mean ± SEM. The data were first tested for normality using the Shipiro–Wilk test. For analysis between two groups, normal data were analyzed with unpaired, 2-tailed *t*-tests while non-normal data were analyzed with the Mann–Whitney U test. For analysis between four groups, two-way ANOVA was used with post hoc Tukey tests where appropriate. All statistics were performed in SPSS (Version 29, IBM Analytics, New York, NY, USA) or R Studio (2024.12.1) and significance was accepted when *p* < 0.05.

## 3. Results

### 3.1. The Effect of a High-Fat Diet on Body Weight and Heart Weight

The effects of 6 months of high-fat feeding on mice body and heart weight are shown in Table 1. High-fat feeding increased the body weight of mice by 18%. However, both the wet heart-to-body weight ratio and the cardiac water content (a measure of oedema) were significantly lower in hearts from high-fat-fed mice.

### 3.2. Effect of High-Fat Diet on Basal Ventricular Metabolite Concentrations

Table 2 shows the baseline (non-ischemic) values for all metabolites measured in ventricular tissue from mice fed a control or high-fat diet. The concentration of hypoxanthine was found to be significantly increased in the high-fat group; otherwise, there were no significant differences in the concentrations of energy-rich and related metabolites (e.g., ATP, AMP, inosine, or adenosine). However, glycogen levels were found to be significantly lower, whereas lactate levels were significantly higher in high-fat diet hearts. High-fat feeding was also associated with a significantly higher concentration of several amino acids: serine, glutamine, histidine, arginine, threonine alanine, and taurine. These differences are consistent with a cardiac metabolic change and/or a shift in substrate utilization in mice fed a high-fat diet.

### 3.3. Effect of High-Fat Diet on Changes in Ventricular Metabolites During I/R

**Figure 2:**
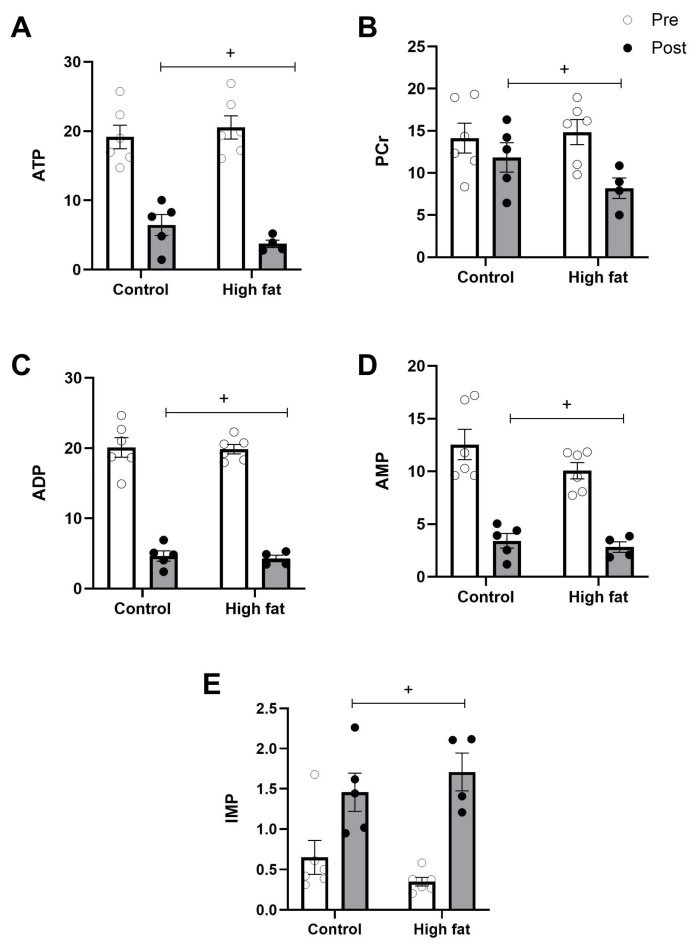
Effect of high-fat diet on changes in cardiac energy-rich phosphate metabolites ((**A**): ATP, (**B**): phosphocreatine, (**C**): ADP, (**D**): AMP, (**E**): IMP) measured before (pre, white bars) and after (post, black bars) I/R injury. Values are mean ± SE (n = 5–6/group). Data were analyzed by two-way ANOVA for the effect of diet (* *p* < 0.05) or time (+ *p* < 0.05). Horizontal bars indicate a significant main effect.

**Figure 3:**
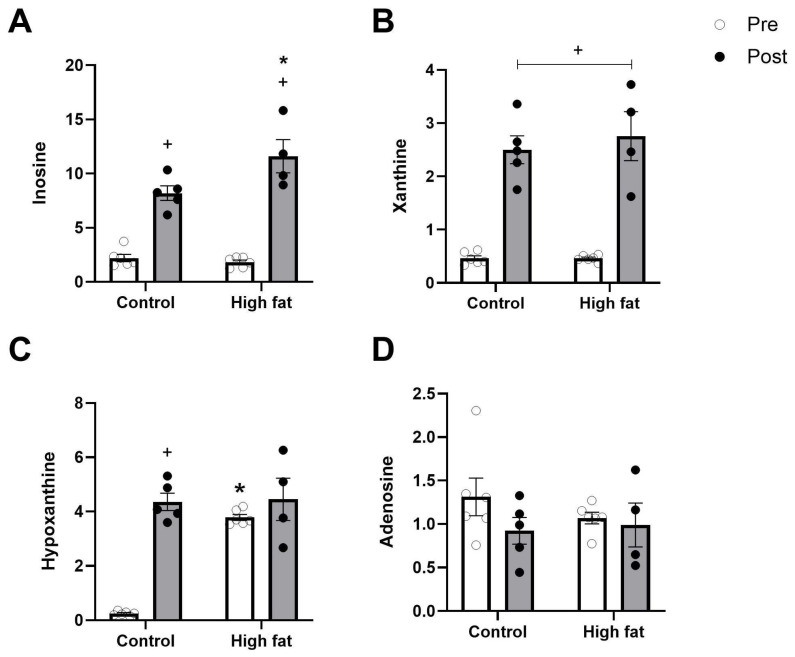
Effect of high-fat diet on changes in cardiac purines ((**A**): inosine, (**B**): xanthine, (**C**): hypoxanthine, (**D**): adenosine) measured before (pre, white bars) and after (post, black bars) I/R injury. Values are mean ± SE (n = 5–6/group). Data were analyzed by two-way ANOVA for the effect of diet (* *p* < 0.05) or time (+ *p* < 0.05). Horizontal bars indicate a significant main effect. Where a significant interaction is observed, the symbols display the outcome of a post hoc Tukey test.

The cardiac tissue concentrations of energy-rich metabolites and purines after I/R are shown in Figure 2 and Figure 3. As expected, I/R was associated with a significant fall in energy-rich phosphates (e.g., ATP, ADP, and AMP) and a rise in related byproducts (e.g., inosine and xanthine) regardless of diet. A fall in PCr and a rise in IMP were also significant regardless of diet (Figure 2B,E). The rise in hypoxanthine between pre and post values was only significant in the control group, however, due to the significantly higher basal level of hypoxanthine in the high-fat group (Figure 3C).

**Figure 4:**
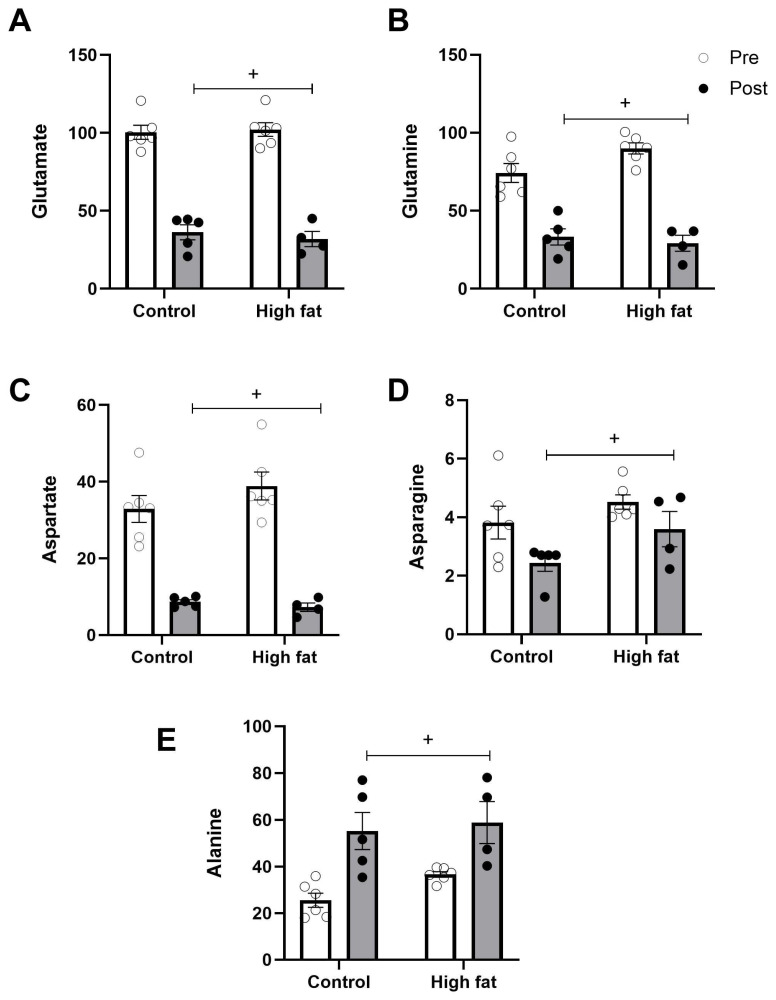
Effect of high-fat diet on changes in cardiac principal protein amino acids ((**A**): glutamate, (**B**): glutamine, (**C**): aspartate, (**D**): asparagine, (**E**): alanine) measured before (pre, white bars) and after (post, black bars) I/R injury. Values are mean± SE (n = 5–6/group). Data were analyzed by two-way ANOVA for the effect of diet (* *p* < 0.05) or time (+ *p* < 0.05). Horizontal bars indicate a significant main effect.

**Figure 5:**
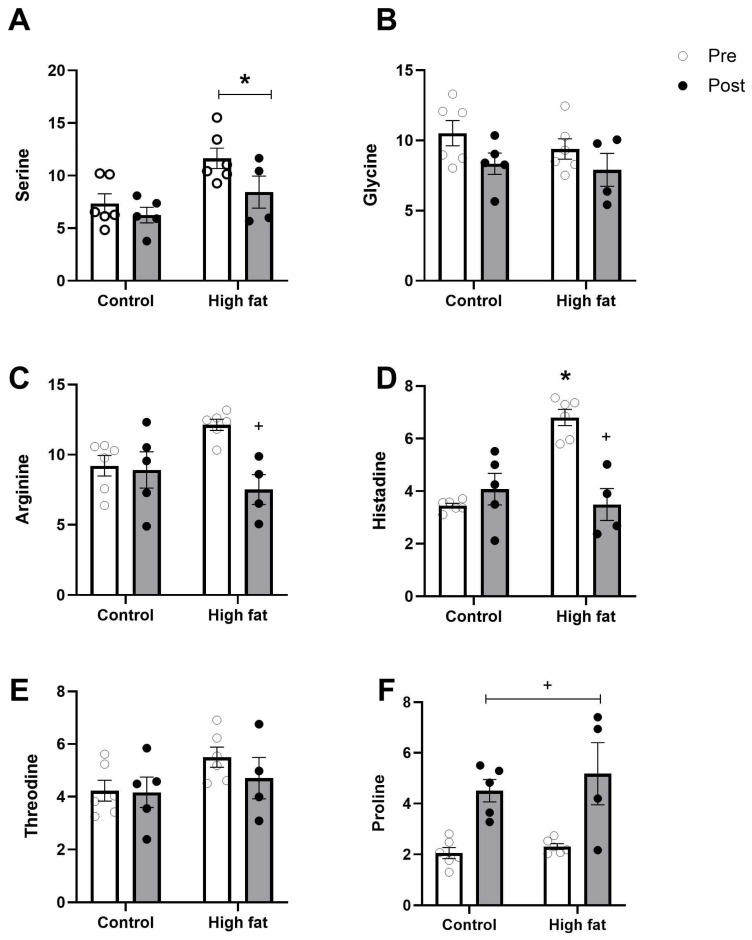
Effect of high-fat diet on changes in other cardiac protein amino acids ((**A**): serine, (**B**): glycine, (**C**): arginine, (**D**): histadine, (**E**): threodine, (**F**): proline) measured before (pre, white bars) and after (post, black bars) I/R injury. Values are mean ± SE (n = 5–6/group). Data were analyzed by two-way ANOVA for the effect of diet (* *p* < 0.05) or time (+ *p* < 0.05). Horizontal bars indicate a significant main effect. Where a significant interaction is observed, the symbols display the outcome of a post hoc Tukey test.

Figure 4 and Figure 5 show changes in the concentrations of amino acids following an I/R challenge. Levels of glutamate, glutamine, and aspartate (the principal protein amino acids that are present at a high concentration) showed a significant fall (regardless of diet) as a result of I/R (Figure 4A–C). In contrast, alanine was significantly increased following I/R (Figure 4E). Some of the amino acids present at a relatively low concentration (e.g., arginine, histidine) showed a significant fall as a result of I/R only in the high-fat diet group (Figure 5C,D).

**Figure 6:**
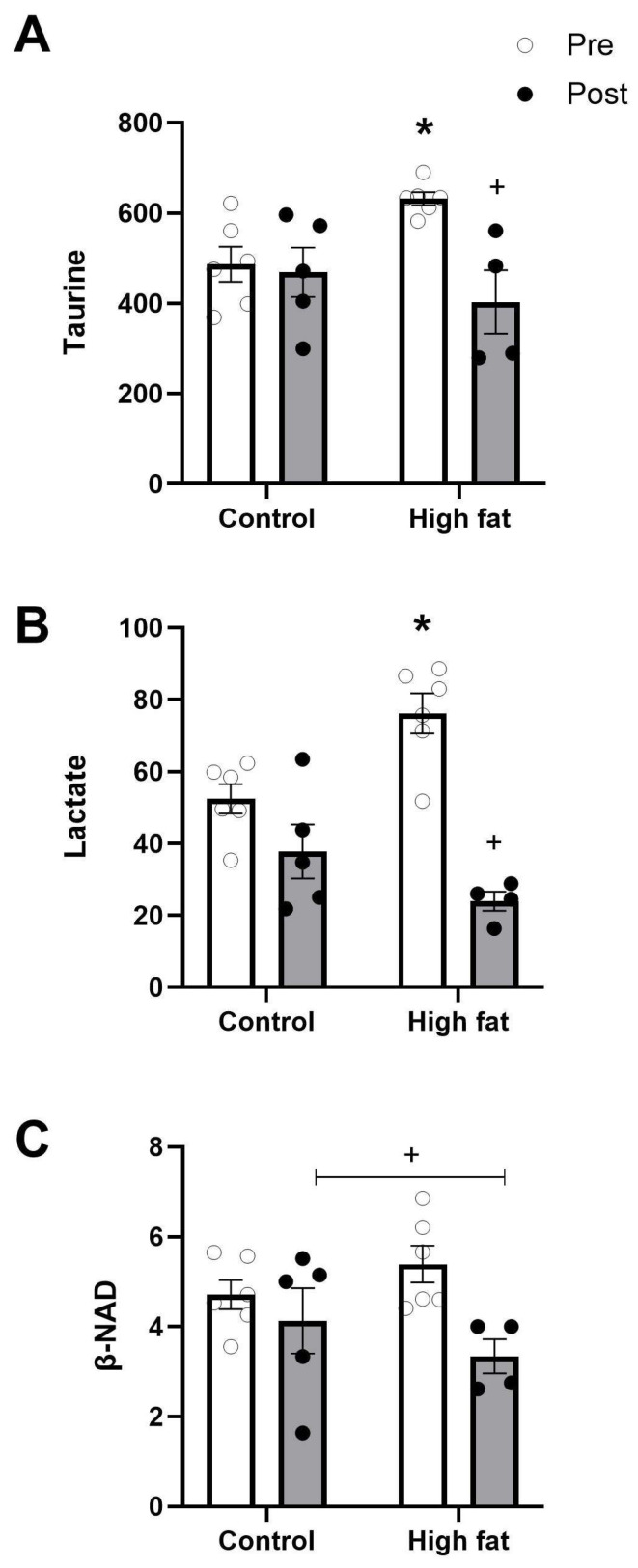
Effect of high-fat diet on markers of osmotic stress (**A**) and metabolic stress (**B**,**C**) measured before (pre, white bars) and after (post, black bars) I/R injury. Values are mean ± SE (n = 5–6/group). Data were analyzed by two-way ANOVA for the effect of diet (* *p* < 0.05) or time (+ *p* < 0.05). Horizontal bars indicate a significant main effect. Where a significant interaction is observed, the symbols display the outcome of a post hoc Tukey test.

Lactate, β-NAD, and taurine are key metabolic stress-related metabolites. The non-protein amino acid taurine, which is present at the highest concentration, did not change during I/R in hearts from the control diet group but showed a marked significant fall in hearts from mice fed a high-fat diet (Figure 6A). Lactate levels were close to basal levels after I/R in control mice hearts but dropped markedly and significantly in high-fat-fed mice (from 76.2 ± 5.6 to 24.0 ± 2.7 nmol/mg protein, Figure 6B). NAD^+^ levels decreased following I/R regardless of diet (Figure 6C). A reduction in NAD^+^ levels during I/R has been used as a marker of mitochondrial permeability transition pore opening [18] and therefore higher vulnerability to I/R.

### 3.4. Effect of High-Fat Diet on Cardiac Ischemic/Metabolic Stress During I/R

**Figure 7:**
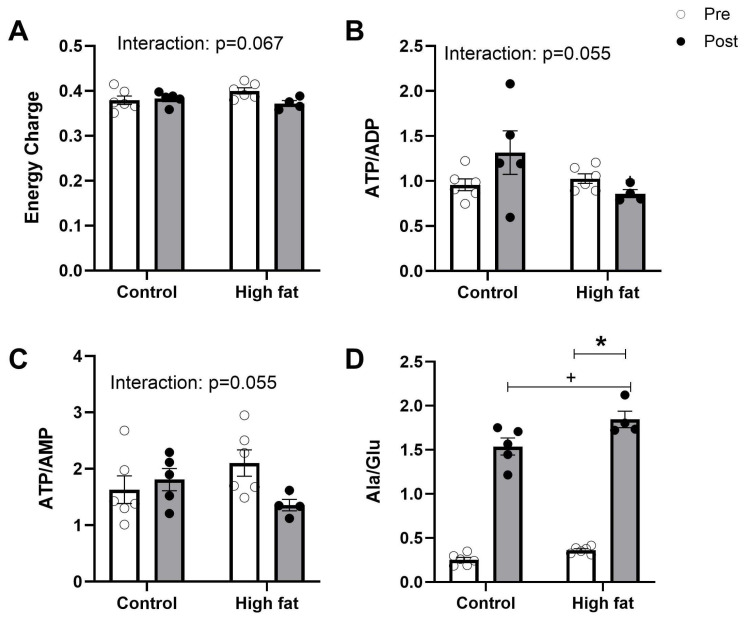
Effect of high-fat diet on computed markers of cardiac energetics (**A**,**B**,**D**) and mitochondrial stress (**C**) measured before (pre, white bars) and after (post, black bars) I/R injury. Values are mean ± SE (n = 5–6/group). Data were analyzed by two-way ANOVA for the effect of diet (* *p* < 0.05) or time (+ *p* < 0.05). Horizontal bars indicate a significant main effect.

In addition to the actual changes in the concentration of individual metabolites during I/R, a number of computed markers were also used to assess the extent of metabolic/ischemic stress in the myocardium during cardiac insults. These are relevant, especially when the concentration of metabolites (energy-rich phosphates and amino acids) appears similar [19]. An example is the alanine/glutamate ratio, which is an indicator of stress that can be evident even when the actual metabolites are not different. Other computed markers, especially those of cardiac energetics (e.g., phosphorylation potential and energy charge), are also important. In this study, energy charge, ATP/ADP, and ATP/AMP all showed trends (not statistically significant but *p* < 0.07) for the interaction between dose and time (Figure 7A–C), and in all cases the values only decreased with time in the high-fat group, suggesting a higher and sustained level of metabolic/ischemic stress during reperfusion. The alanine/glutamate ratio was increased following I/R regardless of diet, and also increased in the high-fat group compared to the control group over both timepoints (Figure 7D).

### 3.5. Effect of High-Fat Diet on Cardiac Pump Function Before and During Ischemia

Our earlier work has shown that hearts from wild-type mice fed an atherogenic (non-obesogenic) high-fat diet are more vulnerable to I/R injury, as measured by impaired function and cardiac injury [4,10]. However, there are no reports on the hemodynamic parameters measured during the ischemic insult which reflect metabolic changes, including time to arrest and to onset of rigor contracture. Table 3 summarizes functional activities before and during ischemia. There were no basal functional differences (heart rate or LVDP) between isolated Langendorff perfused hearts from both groups. Furthermore, the time taken for the hearts to stop beating following ischemic induction was similar. However, the times to onset of rigor contracture and to reach maximal ischemic contracture were significantly shorter for high-fat-diet hearts.

## 4. Discussion

The aim of this study was to identify key cardiac metabolites that could be responsible for the increased vulnerability to I/R in hearts of mice fed a non-obesogenic high-fat diet. This study used a wild-type mouse model of high-fat feeding that is known to preserve cardiac function and is associated with weight gain but no obesity or obesity-related morbidities in wild-type mice. High-fat feeding did not alter the levels of energy-rich phosphates and related metabolites. However, it did trigger the accumulation of several amino acids, including alanine and the non-protein amino acid taurine. Importantly, the myocardium of the high-fat diet group had significantly higher lactate and lower glycogen levels compared to hearts from mice fed a control diet. These changes are consistent with a metabolic change and/or a shift in substrate utilization in mice fed a high-fat diet. While the changes in some of these metabolites have already been shown for hearts from mice fed a high-fat diet, what is not known is the change in these metabolites during I/R that can be used to establish a link to increasing vulnerability to I/R. In this respect, this study demonstrates, for the first time, that high-fat hearts had significantly more changes in markers of ischemic, metabolic, and osmotic stress during I/R compared to hearts from mice fed a high-fat diet. Hyperlipidaemia without obesity therefore predisposes the heart to deleterious metabolic changes that appear to be responsible for the increase in vulnerability to I/R.

### 4.1. Non-Obesogenic, High-Fat Feeding Mouse Model

Mice fed a non-obesogenic high-fat diet are not only resistant to coronary disease, but they also do not readily develop diabetes, hypertension, or hypertrophy unless they are transgenics or undergo further interventions, including extended feeding with a diet that is high in weight-promoting content [4,20,21,22]. A significant increase in body weight of more than 30% is expected in experimental models of obesity in mice [1,2], and the use of sucrose instead of starch in a high-fat diet appears to be a key factor responsible for significant weight gain/obesity [3,4,5]. Although in this study body weight did increase significantly with high-fat feeding, it was not sufficient to be suggestive of obesity in these mice. This could be due to age and the relatively long duration of high-fat feeding. Thus, as indicated earlier on [4], the non-obesogenic high-fat diet used in this study is appropriate for investigating the metabolic effects of hyperlipidaemia on the myocardium to understand the reason for increased vulnerability to I/R.

### 4.2. Chronic Changes in Myocardial Glycogen, Lactate, and Amino Acids, but Not in Energy-Rich Phosphates Due to Feeding a Non-Obesogenic High-Fat Diet

Overall, the basal changes in metabolites due to chronic feeding of a non-obesogenic high-fat diet are indicative of metabolic disruptions and remodeling. For example, high-fat feeding resulted in a significant decrease in basal myocardial glycogen content, and accumulation of both lactate and alanine (Table 2). It is likely that the decreased glycogen content is a result of increased glucose metabolism and utilization. Indeed, there is evidence to suggest that hypercholesterolaemia and excess fatty acids may trigger the myocardium to reactivate the fetal gene expression, resulting in a switch from fat to glucose oxidation [23]. A high-fat diet has also been found to lower the cardiac glycogen content in rats [16,24]. The chronic drop in glycogen could have deleterious consequences as it is an important source of reserve energy used, especially during cardiac insults [25]. In contrast, transient glycogen depletion prior to acute ischemia is cardioprotective as it reduces the dissociation of hexokinase 2 from mitochondria and therefore inhibits the opening of mitochondrial permeability transition pore [26,27,28].

An increase in glycolysis will eventually lead to accumulation of glycolytic byproducts such as lactate and alanine, as shown in this study (Table 2). The increased basal concentration of lactate in the ventricular tissue of high-fat-fed mice also indicates that there is an imbalance in substrate supply versus utilization. This is also reflected by the increased alanine/glutamate ratio in high-fat-fed mice. It is possible that increased lactate (and therefore H^+^) further exacerbates uncoupling of glucose metabolism because of the sensitivity of glycolytic enzymes to pH.

It is widely accepted that lactate (acidosis) and alanine accumulation indicate uncoupling of glycolysis from glucose oxidation (not all glucose could be incorporated into the Krebs cycle), which is expected to be associated with a fall in ATP levels. However, we did not find changes in the basal concentrations of high-energy phosphates (phosphocreatine, ATP, ADP or AMP: Table 2) nor did we see evidence of ischemic stress as measured by EC and ATP/ADP (Figure 7) associated with a high-fat diet. This is consistent with a previous study [4] and the current observation that isolated perfused hearts had a similar developed pressure for both groups (Table 3).

An interesting observation is the finding that a high-fat diet markedly increased basal levels of hypoxanthine, which is an ATP byproduct, known to be elevated when ATP is catabolized during acute ischemic conditions. In this case, the accumulation is chronic due to high-fat feeding. Therefore, it is possible that ATP turnover due to excessive lipid supply has shifted the production of hypoxanthine. This is unlikely to be due to reduced degradation of hypoxanthine as there is no change in xanthine [29].

In addition to alanine, a high-fat diet caused a significant increase in several amino acids (Table 2), including the principal amino acid glutamine, which is present at a high concentration. The intracellular concentration of glutamine was higher at baseline in the high-fat diet. Glutamine can be converted to glutamate to be used as a substrate for the Krebs cycle for energy production during ischemia [30,31]. Since this is measured at basal preischemic conditions, it is possible that this pathway is affected (acidosis associated with lactate). It is likely that the influx of glutamine from extracellular space is responsible.

The reason for the marked increase in the concentration of non-protein amino acid taurine (approximate increase by 20 mmol/L) in response to feeding mice the non-obesogenic high-fat diet is not readily known as this would require time-dependent monitoring and blood level measurements. However, taurine is known to have an antioxidant activity, with the ability to mitigate calcium accumulation [32], which will ensure myocardial integrity. In fact, chronic cardiac taurine deficiency reduces sarcoplasmic reticulum Ca^2+^ ATPase activity and affects function [33]. This could partly explain why hearts of wild-type mice fed a non-obesogenic high-fat diet do not show altered cardiac ROS levels or important functional, structural, and molecular abnormalities [4]. Even though the increase in the level of taurine was marked, it did not seem to increase water content (osmotic swelling, Table 1).

### 4.3. Acute Changes in Cardiac Metabolites During I/R Are More Evident in High-Fat-Diet Hearts and Were Associated with Significant Myocardial Ischemic/Metabolic Stress

Our earlier findings that a high-fat diet without obesity increases vulnerability to I/R injury [4] supports the view that cardiac levels of fatty acids, with or without obesity, directly contribute to the severity of ischemic injury in the heart [34,35,36]. In support of this is the finding that drugs that lower blood lipids reduce arrhythmias and infarct size after I/R [37,38]. However, what is lacking is a link between the degree of metabolic/ischemic stress during I/R and the increased vulnerability emanating from non-obesogenic high-fat feeding. Our data have identified several metabolic pathways (discussed below) that change differently in the high-fat-diet group during I/R, which could underlie this phenomenon.

#### 4.3.1. Cardiac Energetics and Mitochondria

The fall in energy-rich phosphates during ischemia and early reperfusion can be retained during later stages of the reperfusion phase, especially under conditions where the heart has sustained significant injury. Although the fall in energy-rich phosphates occurred in both groups (Figure 2), there were subtle differences, and the effects tended to be more marked in the high-fat group. For example, the fall in PCr was greater in the high-fat group (a fall of approximately 46%) than in the control hearts (a fall of only 16%) (Figure 2B). The fall in ATP was also greater in the high-fat group (an 82% decrease compared to a 66% decrease in the control group). However, a more relevant approach to assess cardiac energetics due to disease and insults is to calculate the phosphorylation potential (ATP/AMP) and energy charge (EC) [19]. EC and phosphorylation potential were not statistically significantly different (Figure 7A,C); however, both showed a strong trend (*p* < 0.07) for significant differences between groups, which in both cases appears to have been driven by a decrease only in the high-fat group, which is consistent with the decreased ability of the heart to produce energy to improve salvage and sustain function. The inability of the cardiomyocytes to deliver the needed energy is likely to involve mitochondrial dysfunction. The fall in β-Nicotinamide adenine dinucleotide (NAD+) at the end of I/R (Figure 6C) occurs regardless of diet but is a larger effect in the high-fat group. Earlier studies have shown that a drop in NAD+ is indicative of opening of the mPTP, which is responsible for cardiomyocyte death [18].

#### 4.3.2. Lactate and Alanine/Glutamate Ratio

Although it is expected that the chronic basal increase in intracellular lactate concentration due to consumption of a high-fat diet may exacerbate acidosis and lead to more severe ionic imbalances and contractile dysfunction, there is no evidence that this is the case in this study. The acute ischemic heart accumulates lactate as it provides a good source of energy [39], but lactate levels are expected to return to basal levels after reperfusion, via metabolism or transport activity. Hearts from mice fed a control diet returned to normal lactate levels at the end of I/R; however in the high-fat group there was a significant fall in lactate levels following I/R, despite expected significant accumulation during ischemia (Figure 6B). The finding that lactate was significantly reduced at the end of I/R in the high-fat group only suggests further/sustained consumption and/or efflux due to the large gradient in the Langendorff heart and possibly due to accumulation of H+ (via the Na/H transporter or anaerobic metabolism). In control hearts, glycogen will provide reserve substrates during I/R. This is not the case in high-fat-fed hearts, where a fatty diet increases mitochondrial fatty acid oxidation, reduces glucose utilization, and results in less glycogenesis. Earlier work has linked glycogen depletion prior to ischemia to cardioprotection by reducing dissociation of hexokinase 2 from mitochondria, and inhibition of the permeability transition pore opening [26,27,28]. These observations were made using acute glycogen depletion, whereas in this case the depletion of glycogen is chronic.

At basal levels, alanine, like lactate, was higher in the high-fat group, which indicates uncoupling of glycolysis from glucose oxidation. Consistent with previous work [30], the alanine/glutamate ratio also markedly increases during I/R, regardless of diet (Figure 7)—the extent of the increase in this ratio is related to I/R injury.

#### 4.3.3. Taurine and Glutamine Loss

The fall in the cardiac level of the slowly metabolized, non-protein β-amino acid taurine seen after I/R in the high-fat group only (Figure 6A) is similar to observations made in patient hearts undergoing open heart surgery using ischemic cardioplegic arrest and reperfusion [31]. The fall in taurine in the latter was linked to increased cardiac metabolic stress and injury. Taurine is known to have the ability to protect cells and can be linked to several factors, including anti-oxidative effects, increases in membrane stability, anti-inflammatory responses, and, importantly for the contractile heart, mitigation of calcium accumulation [32]. However, given the fact that taurine concentration is as high as 40 mM and that it is very slowly metabolized, the acute changes likely reflect the involvement of the taurine/Na^+^ co-transporter. Taurine fluxes are dependent on the Na^+^ gradient across the sarcolemma and heart cells respond to cardiac insults/osmotic stress by releasing amino acids, including taurine [40]. High serum taurine levels in patients after cardiac arrest are significantly associated with higher in-hospital mortality [41], and cardioprotective strategies involve mitigation of taurine loss during I/R [42]. A major cause of ischemia-induced sarcolemmal damage is cell stretching linked to osmotic swelling [43], and it is likely that taurine-loaded cells preferentially remove taurine at the expense of other osmolytes to extrude Na^+^. This study shows that this is the case with a high-fat diet as the gradient is high but does not necessarily protect the heart. It is also likely that the taurine transporter activity is affected by acidosis associated with high lactate levels.

Like taurine, the intracellular concentration of glutamine was higher at baseline in high-fat diet hearts (Table 2). Glutamine concentration was reduced in all animals following I/R, regardless of diet (Figure 4B). This drop in glutamine is likely to be due to the conversion to glutamate to provide the much-needed substrate for the Krebs cycle for energy production during ischemia [31]. It is likely that the transport of glutamine, which is both very fast and Na+-dependent [44], will contribute to the observed fall and effectively suggest that these hearts may have accumulated high levels of Na^+^.

### 4.4. The Changes in Metabolites Due to High-Fat Feeding Alter the Changes in Hemodynamic Parameters During Index Ischemia

The finding that the time to ischemic contracture is similar suggests that the rate of change (fall) in ATP during ischemic arrest is similar for both types of hearts as the starting (resting levels shown in Table 1) concentrations are similar. However, the tendence to onset and maximal ischemic contracture (Table 3) indicate different changes in metabolites and possibly metabolic activity-induced changes in Ca^2+^. Chronic glycogen depletion could be a factor responsible for these differences. Additionally, it is important to note that cardiomyocyte contraction does not only depend on energy availability and Ca^2+^ levels but also on myofilament sensitivity. In fact, there are reports indicating changes in myofilament sensitivity to Ca^2+^ due to exposure to a high-fat diet [45].

### 4.5. Limitations

This study has a number of limitations. One omission is that cardiac glycogen content following reperfusion was not measured. However, basal levels of glycogen are much more informative than post-reperfusion levels, as only changes in basal levels have been shown to be associated with alterations in vulnerability to I/R injury [26,27,28]. The perfusing buffer used was Krebs–Henseleit, which is similar to the circulation of the control animals—while lipids could have been added to the perfusate in the high-fat group, previous studies have demonstrated that vulnerability of hearts to I/R injury is independent of whether the perfusate contains fatty acids or not [16]. The perfusion pressure of 65 mmHg and the LVDP of around 30 mmHg are likely to be lower than the physiological values; however, use of these pressures provides a stable preparation and allows for comparisons with previous studies which have used similar pressures.

## 5. Conclusions

Our study has shown that non-obesogenic high-fat feeding of wild-type mice for 26 weeks does not alter the level of cardiac energy-rich phosphates, energy charge, or phosphorylation potential. However, these hearts have higher levels of taurine, glutamine, alanine, and lactate. Following a period of ischemia and reperfusion (where hearts fed a high-fat diet are known to sustain a significantly higher degree of injury), we show significant differences in markers of ischemic/metabolic, osmotic, and mitochondrial stress in high-fat hearts compared to control hearts. These high-fat diet-induced metabolic changes are independent of disease and predispose these hearts to increased metabolic stress during I/R, which would explain their increased vulnerability. Therefore, implicated metabolic pathways provide potential targets for the design of acute and/or chronic cardioprotective interventions. Ideally, this should target large animal models that will also involve monitoring changes in blood metabolites.

## Figures and Tables

**Table 1:** The effect of high-fat diet on body and heart weight. Values are mean ± SE. * *p* < 0.05 vs. corresponding control diet value (unpaired student’s *t*-test or Mann–Whitney U test, as appropriate).

	Control Diet	High-Fat Diet
	(n = 10)	(n = 11)
Body weight (g)	35.7 ± 1.2	42.2 ± 1.2 *
Heart		
Dry heart weight (mg)	39 ± 2	35 ± 1
Wet heart weight (mg)	209 ± 13	176 ± 8 *
% water content	81.1 ± 0.2	79.8 ± 0.4 *
Wet heart weight/body weight (%)	0.58 ± 0.02	0.42 ± 0.02 *
Cardiac protein content (% wet weight)	10.1 ± 0.7	10.3 ± 0.9

**Table 2:** Myocardial concentration (nmol/mg protein) of all metabolites measured in hearts fed control or high-fat diet. Mean ± SE (n, number of hearts for each group). * *p* < 0.05 vs. corresponding control diet value (unpaired student’s *t*-test or Mann–Whitney U test, as appropriate).

		Control Diet (n = 6)	High-Fat Diet (n = 6)
Energy-rich and related metabolites	**Phosphocreatine**	14.1 ± 1.8	14.9 ± 1.5
	**ATP**	19.2 ± 1.7	20.5 ± 1.7
	**ADP**	20.1 ± 1.4	19.9 ± 0.7
	**AMP**	12.5 ± 1.4	10.1 ± 0.8
	**IMP**	0.65 ± 0.21	0.35 ± 0.05
	**Inosine**	2.2 ± 0.3	1.8 ± 0.2
	**Xanthine**	0.46 ± 0.05	0.46 ± 0.02
	**Hypoxanthine**	0.25 ± 0.03	3.78 ± 0.11 *
	**Adenosine**	1.3 ± 0.2	1.1 ± 0.1
	**B-NAD**	4.7 ± 0.3	5.4 ± 0.4
Protein amino acids	**Aspartate**	32.9 ± 3.5	38.9 ± 3.6
	**Glutamate**	100.4 ± 4.5	102.2 ± 4.4
	**Serine**	7.4 ± 0.9	11.6 ± 1.0 *
	**Asparagine**	3.8 ± 0.6	4.5 ± 0.2
	**Glutamine**	74.2 ± 6.1	90.0 ± 3.5 *
	**Glycine**	10.5 ± 0.9	9.4 ± 0.7
	**Histidine**	3.4 ± 0.1	6.8 ± 0.3 *
	**Arginine**	9.2 ± 0.7	12.1 ± 0.4 *
	**Threonine**	4.2 ± 0.4	5.5 ± 0.4 *
	**Alanine**	25.6 ± 3.0	36.7 ± 1.2 *
	**Proline**	2.1 ± 0.2	2.3 ± 0.1
Non-protein amino acid	**Taurine**	487 ± 39	632 ± 15 *
Other metabolites	**Lactate**	52.5 ± 4.1	76.2 ± 5.6 *
	**Glycogen** (µmol glycosyl units/g wet weight)	126 ± 7	87 ± 12 *

**Table 3:** Haemodynamic functional measurements in Langendorff hearts from mice fed control or high-fat diet before and during 30 min ischemia. Mean ± SE (n, number of hearts for each group). * *p* < 0.05 vs. corresponding control diet value (unpaired student’s *t*-test or Mann–Whitney U test, as appropriate).

	Parameter/Activity	Control Diet (n = 16)	High-Fat Diet (n = 13)
Haemodynamic parameters pre-ischemia	LVDP (mmHg)	31.5 ± 3.2	31.8 ± 3.6
	Heart rate (bpm)	394 ± 20	417 ± 19
Changes during ischemia	Time to arrest (min)	3.5 ± 0.3	3.8 ± 0.3
	Time to onset of rigor contracture (min)	15.5 ± 1.0	13.0 ± 0.8 (*p* = 0.050)
	Time to max ischemic contracture (mins)	22.1 ± 0.8	17.5 ± 0.9 *
	Maximum end diastolic pressure (mmHg)	42.0 ± 4.1	45.7 ± 5.5

## Data Availability

The raw data supporting the conclusions of this article will be made available by the authors upon request.

## Abbreviations

The following abbreviations are used in this manuscript:

HPLC	High-performance liquid chromatography
I/R	Ischemic reperfusion

## References

[1] Calligaris S.D., Lecanda M., Solis F., Ezquer M., Gutierrez J., Brandan E., Leiva A., Sobrevia L., Conget P. (2013). Mice long-term high-fat diet feeding recapitulates human cardiovascular alterations: An animal model to study the early phases of diabetic cardiomyopathy. PLoS ONE.

[2] Noyan-Ashraf M.H., Shikatani E.A., Schuiki I., Mukovozov I., Wu J., Li R.K., Volchuk A., Robinson L.A., Billia F., Drucker D.J. (2013). A glucagon-like peptide-1 analog reverses the molecular pathology and cardiac dysfunction of a mouse model of obesity. Circulation.

[3] Hallfrisch J., Cohen L., Reiser S. (1981). Effects of feeding rats sucrose in a high fat diet. J. Nutr..

[4] Littlejohns B., Pasdois P., Duggan S., Bond A.R., Heesom K., Jackson C.L., Angelini G.D., Halestrap A.P., Suleiman M.S. (2014). Hearts from Mice Fed a Non-Obesogenic High-Fat Diet Exhibit Changes in Their Oxidative State, Calcium and Mitochondria in Parallel with Increased Susceptibility to Reperfusion Injury. PLoS ONE.

[5] Surwit R.S., Kuhn C.M., Cochrane C., McCubbin J.A., Feinglos M.N. (1988). Diet-induced type II diabetes in C57BL/6J mice. Diabetes.

[6] Toida S., Takahashi M., Shimizu H., Sato N., Shimomura Y., Kobayashi I. (1996). Effect of high sucrose feeding on fat accumulation in the male Wistar rat. Obes. Res..

[7] Gallou-Kabani C., Vige A., Gross M.S., Rabes J.P., Boileau C., Larue-Achagiotis C., Tome D., Jais J.P., Junien C. (2007). C57BL/6J and A/J mice fed a high-fat diet delineate components of metabolic syndrome. Obesity (Silver Spring).

[8] Williams T.D., Chambers J.B., Roberts L.M., Henderson R.P., Overton J.M. (2003). Diet-induced obesity and cardiovascular regulation in C57BL/6J mice. Clin. Exp. Pharmacol. Physiol..

[9] Mills E., Kuhn C.M., Feinglos M.N., Surwit R. (1993). Hypertension in CB57BL/6J mouse model of non-insulin-dependent diabetes mellitus. Am. J. Physiol..

[10] Chase A., Jackson C.L., Angelini G.L., Suleiman M.S. (2007). Coronary artery disease progression is associated with increased resistance of hearts and myocytes to cardiac insults. Crit. Care Med..

[11] Liu J., Wang P., Douglas S.L., Tate J.M., Sham S., Lloyd S.G. (2016). Impact of high-fat, low-carbohydrate diet on myocardial substrate oxidation, insulin sensitivity, and cardiac function after ischemia-reperfusion. Am. J. Physiol. Heart Circ. Physiol..

[12] Liu J., Wang P., Zou L., Qu J., Litovsky S., Umeda P., Zhou L., Chatham J., Marsh S.A., Dell’Italia L.J. (2014). High-fat, low-carbohydrate diet promotes arrhythmic death and increases myocardial ischemia-reperfusion injury in rats. Am. J. Physiol. Heart Circ. Physiol..

[13] Liu J., Lloyd S.G. (2013). High-fat, low-carbohydrate diet alters myocardial oxidative stress and impairs recovery of cardiac function after ischemia and reperfusion in obese rats. Nutr. Res..

[14] Panagia M., Gibbons G.F., Radda G.K., Clarke K. (2005). PPAR-alpha activation required for decreased glucose uptake and increased susceptibility to injury during ischemia. Am. J. Physiol. Heart Circ. Physiol..

[15] Lloyd-Jones D.M., Evans J.C., Larson M.G., O’Donnell C.J., Wilson P.W., Levy D. (1999). Cross-classification of JNC VI blood pressure stages and risk groups in the Framingham Heart Study. Arch. Intern. Med..

[16] Wang P., Tate J.M., Lloyd S.G. (2008). Low carbohydrate diet decreases myocardial insulin signaling and increases susceptibility to myocardial ischemia. Life Sci..

[17] Imura H., Caputo M., Parry A., Pawade A., Angelini G., Suleiman M. (2001). Age-dependent and hypoxia-related differences in myocardial protection during pediatric open heart surgery. Circulation.

[18] Di Lisa F., Menabo R., Canton M., Barile M., Bernardi P. (2001). Opening of the mitochondrial permeability transition pore causes depletion of mitochondrial and cytosolic NAD+ and is a causative event in the death of myocytes in postischemic reperfusion of the heart. J. Biol. Chem..

[19] Abdul-Ghani S., Fleishman A.N., Khaliulin I., Meloni M., Angelini G.D., Suleiman M.S. (2017). Remote ischemic preconditioning triggers changes in autonomic nervous system activity: Implications for cardioprotection. Physiol. Rep..

[20] Paigen B., Morrow A., Holmes P.A., Mitchell D., Williams R.A. (1987). Quantitative assessment of atherosclerotic lesions in mice. Atherosclerosis.

[21] Paigen B., Morrow A., Brandon C., Mitchell D., Holmes P. (1985). Variation in susceptibility to atherosclerosis among inbred strains of mice. Atherosclerosis.

[22] Breslow J.L. (1996). Mouse models of atherosclerosis. Science.

[23] Wong C., Marwick T.H. (2007). Alterations in myocardial characteristics associated with obesity: Detection, mechanisms, and implications. Trends Cardiovasc. Med..

[24] Diniz Y.S., Cicogna A.C., Padovani C.R., Santana L.S., Faine L.A., Novelli E.L. (2004). Diets rich in saturated and polyunsaturated fatty acids: Metabolic shifting and cardiac health. Nutrition.

[25] Henning S.L., Wambolt R.B., Schonekess B.O., Lopaschuk G.D., Allard M.F. (1996). Contribution of glycogen to aerobic myocardial glucose utilization. Circulation.

[26] Khaliulin I., Parker J.E., Halestrap A.P. (2010). Consecutive pharmacological activation of PKA and PKC mimics the potent cardioprotection of temperature preconditioning. Cardiovasc. Res..

[27] Pasdois P., Parker J.E., Halestrap A.P. (2013). Extent of mitochondrial hexokinase II dissociation during ischemia correlates with mitochondrial cytochrome c release, reactive oxygen species production, and infarct size on reperfusion. J. Am. Heart Assoc..

[28] Lewis M., Szobi A., Balaska D., Khaliulin I., Adameova A., Griffiths E., Orchard C.H., Suleiman M.S. (2018). Consecutive Isoproterenol and Adenosine Treatment Confers Marked Protection against Reperfusion Injury in Adult but Not in Immature Heart: A Role for Glycogen. Int. J. Mol. Sci..

[29] Lopez-Schenk R., Collins N.L., Schenk N.A., Beard D.A. (2023). Integrated Functions of Cardiac Energetics, Mechanics, and Purine Nucleotide Metabolism. Compr. Physiol..

[30] Skeffington K.L., Moscarelli M., Abdul-Ghani S., Fiorentino F., Emanueli C., Reeves B.C., Punjabi P.P., Angelini G.D., Suleiman M.S. (2022). Pathology-related changes in cardiac energy metabolites, inflammatory response and reperfusion injury following cardioplegic arrest in patients undergoing open-heart surgery. Front. Cardiovasc. Med..

[31] Suleiman M.S., Moffatt A.C., Dihmis W.C., Caputo M., Hutter J.A., Angelini G.D., Bryan A.J. (1997). Effect of ischaemia and reperfusion on the intracellular concentration of taurine and glutamine in the hearts of patients undergoing coronary artery surgery. Biochim. Biophys. Acta.

[32] Baliou S., Adamaki M., Ioannou P., Pappa A., Panayiotidis M.I., Spandidos D.A., Christodoulou I., Kyriakopoulos A.M., Zoumpourlis V. (2021). Protective role of taurine against oxidative stress (Review). Mol. Med. Rep..

[33] Ramila K.C., Jong C.J., Pastukh V., Ito T., Azuma J., Schaffer S.W. (2015). Role of protein phosphorylation in excitation-contraction coupling in taurine deficient hearts. Am. J. Physiol. Heart Circ. Physiol..

[34] Stanley W.C., Lopaschuk G.D., Hall J.L., McCormack J.G. (1997). Regulation of myocardial carbohydrate metabolism under normal and ischaemic conditions. Potential for pharmacological interventions. Cardiovasc. Res..

[35] Lopaschuk G.D. (2002). Metabolic abnormalities in the diabetic heart. Heart Fail. Rev..

[36] Pepe S., McLennan P.L. (2002). Cardiac membrane fatty acid composition modulates myocardial oxygen consumption and postischemic recovery of contractile function. Circulation.

[37] Harper C.R., Jacobson T.A. (2001). The fats of life: The role of omega-3 fatty acids in the prevention of coronary heart disease. Arch. Intern. Med..

[38] Adameova A., Kuzelova M., Faberova V., Svec P. (2006). Protective effect of simvastatin and VULM 1457 in ischaemic-reperfused myocardium of the diabetic-hypercholesterolemic rats. Pharmazie.

[39] Dong S., Qian L., Cheng Z., Chen C., Wang K., Hu S., Zhang X., Wu T. (2021). Lactate and Myocardiac Energy Metabolism. Front. Physiol..

[40] Schaffer S.W., Solodushko V., Kakhniashvili D. (2002). Beneficial effect of taurine depletion on osmotic sodium and calcium loading during chemical hypoxia. Am. J. Physiol. Cell Physiol..

[41] Herzog N., Laager R., Thommen E., Widmer M., Vincent A.M., Keller A., Becker C., Beck K., Perrig S., Bernasconi L. (2020). Association of Taurine with In-Hospital Mortality in Patients after Out-of-Hospital Cardiac Arrest: Results from the Prospective, Observational COMMUNICATE Study. J. Clin. Med..

[42] Justice C.N., Zhu X., Li J., O’Donnell J.M., Vanden Hoek T.L. (2023). Intra-ischemic hypothermia cardioprotection involves modulation of PTEN/Akt/ERK signaling and fatty acid oxidation. Physiol. Rep..

[43] Schaffer S.W., Jong C.J., Ito T., Azuma J. (2014). Effect of taurine on ischemia-reperfusion injury. Amino Acids.

[44] Rennie M.J., Tadros L., Khogali S., Ahmed A., Taylor P.M. (1994). Glutamine transport and its metabolic effects. J. Nutr..

[45] Howarth F.C., Qureshi M.A., Gbewonyo A.J., Tariq S., Adeghate E. (2005). The progressive effects of a fat enriched diet on ventricular myocyte contraction and intracellular Ca^2+^ in the C57BL/6J mouse. Mol. Cell. Biochem..

